# Work-Related Factors Considered by Sickness-Absent Employees When Estimating Timeframes for Returning to Work

**DOI:** 10.1371/journal.pone.0163674

**Published:** 2016-10-05

**Authors:** Amanda E. Young, YoonSun Choi

**Affiliations:** Center for Disability Research, Liberty Mutual Research Institute for Safety, Hopkinton, Massachusetts, United States of America; Chang Gung Memorial Hospital Kaohsiung Branch, TAIWAN

## Abstract

**Introduction:**

Work-related factors have been found to be influential in shaping a number of return-to-work outcomes including return-to-work expectations. Based on the idea that work-related factors have the potential for modification through workplace-based initiatives, this study involved a detailed examination of work-related factors referenced by workers as being taken into consideration when estimating timeframes for returning to work.

**Methods:**

Focus groups were conducted with 30 employees, currently off work (≤ 3 months) due to a musculoskeletal condition. During the focus groups, participants wrote and spoke about the factors that they considered when forming their expectations for returning to work. Data were subjected to thematic content analysis.

**Results:**

Discussions revealed that participants’ considerations tended to differ depending on whether or not they had a job to return to. Those with jobs (n = 23) referenced specific influences such as working relationships, accommodations, physical and practical limitations, as well as concerns about their ability to do their job. Those without a job to return to (n = 7) talked about the ways they would go about finding work, and how long they thought this would take. Both groups mentioned the influence of wanting to find the “right” job, retraining and being limited due to the need for income.

**Conclusion:**

Findings indicate that employees reference numerous work-related factors when estimating their timeframes for returning to work, and that many of these have been previously identified as relating to other return-to-work outcomes. Findings suggest the potential to improve return-to-work expectation through addressing work-related influences, and helping people work through the tasks they need to complete in order to move forward in the return-to-work process.

## Introduction

Expectations have been found to relate to outcomes in many domains of human existence. They have been found to have an important impact on decision-making in a wide variety of consumer settings [1]. They are important predictors of the outcome of analgesic treatments [2]. Expectations have been identified as a predictor of outcome in non-chronic non-specific low back pain [3]. And more specific to the current topic, workers’ own expectations for returning to work have been found to be one of the best predictors of actual return to work outcomes [4–7]. Expectations and injury perceptions are increasingly being explored for their relationship to work disability because of their role in influencing behaviors [8]. Although there is the understanding that return-to-work expectations are related to outcomes, the reason for this is still unclear. Researchers have suggested that individuals assess a myriad of physical, personal and environmental variables when forming their return-to-work expectations [9]. Research exploring how people determine their recovery expectations identified the importance of self (beliefs, attitudes and past experiences), pain (perceptions, as a barrier to activity and attitude towards pain), progression (improvement and getting worse) and performance (including usual activities of daily living and at work) [3]. However, there had been little research that had sought to gain an understanding of the factors people reference when asked to make predictions about their future return to work.

Based on the idea that, if we can improve the understanding of return-to-work expectation formation, we will be better placed to develop work-disability interventions, we conducted a study of what people consider when forming their expectations for returning to work. The current report focuses on a detailed examination of work-related influences. Our rationale for this is that we found work-related factors to be referenced as some of the most influential in shaping return-to-work expectations [10] and we surmised that these have a high potential for modification through workplace-based initiatives aimed at improving return-to-work outcomes.

Although there has not been research into the role of work-related factors in forming expectations for returning to work, past research does provide insight. A systematic review found moderate evidence that changes in work schedules and work organization, along with flexibility of work schedules and support from the employer, promote employment among physically-disabled persons [11]. Flexibility of working conditions in terms of hours, duties, equipment, breaks, and pace have been identified as employer-based facilitators of return to work following a disabling injury [12, 13]. Union representation [14], positive relationships with one’s supervisor [15, 16] as well as receiving social support from co-workers [13, 17, 18], the employer [12], or both [19] have also been found to be associated with positive return-to-work outcomes. Another systematic review identified employment variables associated with reduced labor market desirability as a predictor of poor return-to-work outcomes following workplace injury [20]. Similarly, it has been found that the worker’s value to the employer and the nature of the job (e.g., availability of suitable work adjustments) can influence the outcomes of the return-to-work process: “low-value” workers at workplaces with limited possibilities to offer workplace adjustments are at high risk for dismissal [21]. Additionally, the employer’s doubt of the work-relatedness of the worker’s injury was predictive of prolonged time on benefits for injured workers with acute back pain [14].

To summarize, although research has demonstrated that work-related factors influence return-to-work outcomes, it is not known if such factors are considered by employees when they estimate their timeframe for returning to work following sickness-related absence. In order to gain an understanding of what might be done at the workplace to improve the return-to-work outcomes, we conducted a study to identify work-related factors that employees who are off work due to a health condition often consider when estimating their timeframe for returning to work.

## Method

This report contains an analysis of data collected as part of a larger study investigating factors taken into consideration when forming expectations for returning to work. In an earlier report, we outlined the seven main themes as they related to the identified contexts (i.e. self, condition, disability management, *work*, social, physical, and economic) [10]. This paper focuses on the prominent influences within the *work* context.

### Study Design

Qualitative description was chosen to address our research question [22, 23]. This approach was deemed appropriate given that our aim was to describe the work-related influences people take into consideration when forming their expectations for returning to work. Qualitative description involves a rich, straight description of an experience or an event [24]. As such, we saw it as the most appropriate approach for addressing our research question. Focus groups were used as the mechanism for data collection for two reasons. First, it was felt that having a conversation with others in a similar situation would enable free expression and storytelling. Second, focus groups allow the researchers to hear participants’ metaphors for their problems and to gain a better understanding of the context [25]. A purposive sampling strategy was used to select participants based on their eligibility and ability to attend a focus group session.

### Procedures

A detailed description of our procedures and analytical method has been described previously [10]. To summarize, advertisements in local newspapers and digital media were used to recruit participants. To be eligible to participate in the research, participants had to be of working age, working at least 35 hours a week at the time of injury, and currently off work due to a musculoskeletal condition but for no longer than three months. For this analysis, we also excluded people who were self-employed due to the injured worker’s dual role of worker and employer.

Focus groups were held between February and August, 2013, at our research facility located in the greater Boston area, Massachusetts, USA. No efforts were made to compose groups based on socio-demographic characteristics. The groups were facilitated by a total of three (two per data collection session) female researchers with experience in conducting qualitative research. Although all groups were scheduled to include two facilitators and a minimum of three participants, in three instances “no shows” resulted in only one participant being present. Rather than rescheduling, the two facilitators interviewed the sole attendee.

Prior to the commencement of the focus group, each participant completed the informed consent process. All procedures followed were in accordance with the Helsinki Declaration of 1975 as revised in 2000, and the study was approved by the New England Institutional Review Board. Once consent had been received, each participant completed a questionnaire inquiring about: (i) whether they expected to return to work (yes/no), (ii) if so, the approximate timeframe for doing so, and (iii) the factors they considered when forming their responses. Participants were instructed to list as many influences as they wanted to. A full lined page was provided.

After the collection of completed questionnaires, participants were asked to briefly introduce themselves. The lead facilitator then went through the influences listed by the participants, asking the relevant participants to expand on their responses, and then asking others to share similar or contrasting experiences. Throughout the discussions, the lead facilitator checked in with participants regarding her understanding of what was being discussed. This involved the facilitator summarizing what people had been talking about and asking the group if the summary was accurate. Each data collection session was approximately two hours in duration. Focus groups were audio-recorded and notes were also taken.

After the completion of each group, the facilitators met to debrief and code observations. A record was kept of “newly” reported considerations. Sampling continued until the facilitators felt as though they were not accessing new information (i.e. saturation had been reached). As reported in our earlier paper [10], post-hoc analysis indicated that our assessment was correct. Audio-recordings were transcribed verbatim by an experienced transcriber.

### Analysis

Questionnaire responses, audio recordings, and field notes were subjected to thematic content analysis. The process of analyzing the data involved several iterative steps. First, we reviewed the transcripts to gain a sense of emerging themes. Then, we began the process of coding the focus group transcripts. Rather than assigning *a priori* codes, two researchers independently reviewed the data and identified thematic content from within the data. Consistent with the qualitative description approach [22, 23], response codes were counted with the aim of identifying patterns and regularities in the data. As previously described, we found that much of what people spoke about could be compartmentalized to reflect features of themselves, their condition, or their broader environmental contexts (work, social, physical and economic) [10]. For the current paper, we conducted a more detailed analysis of responses referencing the “work” context. To achieve this, we used NVivo 10 (QSR International) to identify data that had been coded as referencing work. To identify themes, we reviewed the tagged data to identify the work-related factors cited as influencing the participant’s expected timeframe for returning to work. Once influencing factors had been identified, they were analyzed for their relationship to return to work. This involved determining if the factor was moving the individual towards (catalytic) or away from (inhibitory) returning to work.

### Participants

Of the 145 people who expressed an interest in participating, 68 people were eligible; of these, 34 participated in the research and 8 more, although scheduled, did not attend. The remaining 26 persons were not sampled as it was felt that saturation had been achieved. Participants’ ages ranged from 24 to 65 years old (*M* = 44, *SD* = 13, n = 30). People were off work for a variety of conditions including back pain (n = 12, 40.0%), upper limb injury (n = 10, 33.3%), lower limb injury (n = 7, 23.3%) and chronic pain (n = 1, 3.3%). All had been out of work for 3 months or less due to their work-disabling musculoskeletal condition (*M* = 50 days, *SD* = 21, Range = 14–90 days). Most had a work-related injury (n = 23, 76.7%); however, only 10 (33.3%) had filed a Workers’ Compensation claim. Most participants (63.3%) held physically demanding jobs. At the time of participating in the focus groups, all participants were off work and reported expecting to return to work. Most commonly, participants expected to return to work within 30 days from the date of the focus group (*M* = 46 days, *SD* = 49, Range = 1 day-6 months).] See Table 1 for more details regarding participant characteristics and predicted timeframe for returning to work.

**Table 1:** Participant (N = 30) characteristics and expected timeframe for returning to work.

ID# ^ǂ^	Gender	Age	Occupation	Off Work Due To	Work-Related	WC Claim Filed	Job to Return To	Estimated Time to RTW
FG1.1	M	57	Office worker	Hip injury	Yes	Yes	Yes	6 weeks
FG1.2	M	35	Sales professional	Knee injury	Yes	Yes	Yes	10 days
FG2.1	M	63	Maintenance	Shoulder injury	Yes	Yes	No	6–12 weeks
FG2.2	M	65	Driver	Back pain	Yes	Yes	Yes	1 month
FG3.1	F	49	Patient care assistant	Back pain	Yes	No	Yes	2 weeks
FG3.3	M	47	Carpenter	Fractured toes	Yes	Yes	Yes	1 week
FG4.2	M	43	Office worker	Back pain	No	No	Yes	2 weeks
FG4.3	F	50	Patient care assistant	Back pain	No	No	Yes	12–19 days
FG4.4	M	27	Mechanic	Fractured elbow	No	No	Yes	6 days
FG5.2	F	36	Restaurant service	Back pain	Yes	No	Yes	11 days
FG5.3	M	49	Studio assistant	Back pain	Yes	No	Yes	15 days
FG6.1	M	29	Construction worker	Back pain	Yes	No	No	3–9 months
FG6.2	M	52	Shop assistant	Back pain	No	No	Yes	2 weeks
FG6.3	M	34	Restaurant service	Hand injury	Yes	No	Yes	1 day
FG7.1	F	34	Office worker	Chronic pain	No	No	No	3–4 weeks
FG8.1	M	29	Landscaper	Knee injury	Yes	Yes	Yes	6 months
FG9.1	M	24	Office worker	Wrist pain	Yes	No	No	3–6 months
FG9.2	M	28	Landscaper	Back strain	Yes	No	Yes	1–2 weeks
FG10.1	M	25	Mechanic	Back pain	Yes	No	Yes	3 months
FG10.2	F	60	Office worker	Hand pain	No	No	Yes	4–5 weeks
FG10.3	M	31	Construction worker	Back pain	Yes	No	No	3 months
FG10.4	M	63	Construction worker	Shoulder injury	Yes	Yes	Yes	10 weeks
FG10.5	M	50	Office worker	Shoulder injury	Yes	No	Yes	2–3 weeks
FG11.1	M	50	Nurse aide	Fractured elbow	No	No	No	3 months
FG11.2	F	55	Patient care assistant	Leg pain, Knee pain	Yes	No	No	3 months
FG11.3	M	35	Massage therapist	Hand sprain	Yes	Yes	Yes	10 days
FG12.1	M	52	Office worker	Knee sprain	Yes	No	Yes	2–6 weeks
FG13.1	M	55	Driver	Back pain	Yes	Yes	Yes	1 month
FG13.2	M	59	Sales professional	Back pain	Yes	Yes	Yes	4 weeks
FG14.1	M	30	Teacher	Foot pain	Yes	No	Yes	2 weeks

^ǂ^ Identification numbers (ID#) indicate the focus group in which the individual participated, and his or her number within the group (e.g. FG1.1 = Focus Group 1, Participant 1).

RTW = Return to work.

## Results

**Fig 1:**
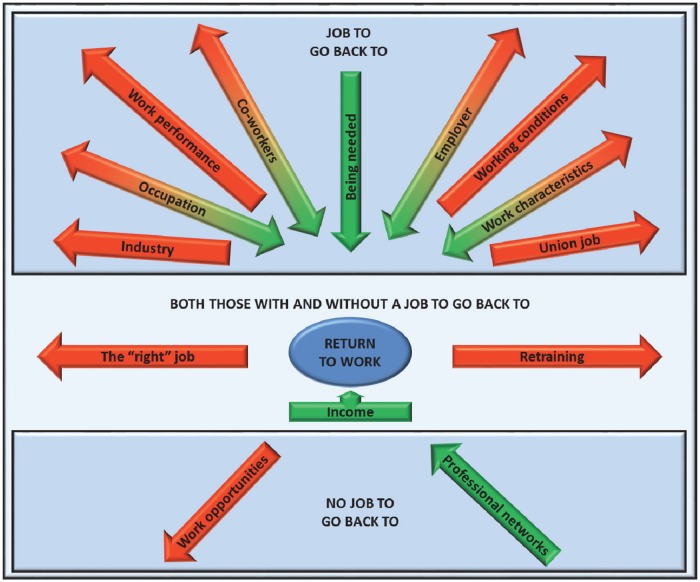
Work-related factors mentioned as influencing return-to-work expectations for sickness-absent employees with musculoskeletal conditions. Arrows indicate the general nature of the influence; that is, moving the individual towards or away from returning to work. Bi-directional arrows indicate influences that were said to move some participants towards returning to work and others away from it.

Group discussions revealed that participants’ considerations tended to differ depending on whether or not they had a job to return to. Those with jobs (n = 23) referenced specific influences such as working relationship, accommodations, physical and practical limitations, as well as concerns about their ability to do their job. Those without a job to return to (n = 7) talked about the ways they would go about finding one and how long they thought this would take. Both those with a job to go back to and those without mentioned the influence of wanting to find the “right” job, retraining and being limited due to the need for income. A summary of the work-related influences mentioned and the number of people who referenced them is contained in Table 2. Fig 1 summarizes the main themes and the direction of the influence (i.e. moving the individual towards or away from returning to work). These are discussed in greater detail below.

**Table 2:** Summary of work-related influences mentioned by participants (N = 30) when asked about what they took into consideration when estimating their return to work.

Work-Related Influence	Participants Mentioning Influence (N = 30)	Elaboration
Employer / Supervisor	23	Employer or supervisor relationship, and the worker’s perceptions of their willingness to accommodate limitations.
Co-workers	12	Perceptions of the reactions of workplace peers and colleagues.
Being needed	5	Perceptions of feeling needed at work by employer, supervisor, co-worker(s), and/or clientele.
Work performance	4	Perceptions of being able to perform the work to an acceptable standard.
Work characteristics	12	Duties, hours, workplace flexibility (hours and/or duties).
Physical working conditions	17	Physical environment (e.g., fumes, temperature), climatic/weather conditions (e.g., snow, ice, rain), and availability of equipment.
Occupation / Industry	12	Seasonal nature of job. Whether light duties are possible/available.
Union job	4	Whether or not the participant was a member of a union, or had union representation.
Work opportunities	11	Time needed to find work. Work opportunities tended to be viewed as limited due to health condition, the economy and age.
Professional networks	3	Time needed to attend networking events, reconnecting with former colleagues and other acquaintances to identify job opportunities.
The “right” job	8	Time to find a good match between their capabilities and job demands.
Retraining	5	Time to complete additional education, learning job skills, or new training in a different line of work.
The need for income	20	Need to return due to need for income. Other resources running out.

### Job to go back to

For those with a job to go back to (n = 23), spoken-about influences effecting RTW expectations were multifaceted and could produce either a catalytic or inhibitory effect. Prominent were workplace relationships, perceptions of performance and judgment.

#### Employer / supervisor

A number of the study participants spoke about how their relationship with their employer would mean that they had a job to go back to.

My boss is really good… uh, nice about it. They, like, um, “Come back when you’re ready,” you know? So. Okay, fine with me. “I NEED you, but come back when you’re ready.” Because I’m working with food, you know, so. They’re not gonna want to take it too fast or something like that, so, “Take your time,” and, you know?”

(FG10.1)

I’m not worried that I have to sit out, because I know I have my desk and the office left. They’ll be there when I get back. Now, if I was more worried about, you know, going back to work and there’s somebody sitting in my office, and, “who are you?”, then I’d be more worried about it. But I’m not.

(FG1.1)

However, the influence of the employer was not always facilitatory of an optimal return-to-work experience. Participants spoke about feeling pressure to go back to work before they might otherwise be ready, and about how feelings of being treated fairly influenced their RTW estimations.

Basically, I’m planning to go back to work in two weeks. I probably won’t be ready, but, I feel guilty, as I’ve known [my boss] for fifteen years, and I feel obligated to go back and help him.

(FG5.3)

I just want to be made right. I am out of work because something happened at work. I think if they were to really pressure [me], maybe start discounting what happened, then I would maybe take a stand back.

(FG1.2)

#### Co-workers

As was the case for the influence of employers, we found that depending on the circumstances, co-workers could exert both a catalytic and inhibitory force on RTW expectations. On the positive side, participants spoke of wanting to get back to work because they missed their coworkers.

[I miss my] friends at work. Just the people that you’re friendly with at work, you know? I’ve got some buddies at work and stuff, so, stuff like that.

(FG4.2)

However, there were also participants who expressed concerns about fitting back into the workplace, and that this influenced their RTW estimates.

I’m not really sure what to expect, going back to work. I’ve got a few concerns, trying to fit back in with the crew. A lot of testosterone running around and everyone’s very competitive.

(FG3.3)

Some urgency for return to work could be seen in responses indicating that participants were worried about the potential for co-workers taking their jobs. This urgency tended to be associated with RTW expectations that were less closely aligned with condition recovery.

You know, I’m important that day because I’m doing my job. But if I don’t ever come back for some reason, Joe Schmoe’s going to come in there and he’s going to take over. So, none of us are, you know, indispensable. […] And now I have a 19-year old kid in there [my office], and now he’s changed a bunch of things […]. He can, you know, he can do my day off, but, that’s enough. I want my job. I like what I do!

(FG10.2)

#### Being needed

Perceptions regarding being needed at the workplace also influenced expected timeframes for returning to work. We found that feeling needed was catalytic of desire for sustained return to work, but not necessarily for the most expedient return to work.

Most of it [desire to return] is because of my students and their families. I’ve heard so many stories and testimonials from parents and students alike which have touched me to the point where I’m just like, I HAVE to be here! […] Earlier, I was younger and more about myself. And that’s when I kind of didn’t take care of it [health condition]. And now that I’m thinking of other people… and how important it is to be there for others, I want to be at my best, in order to do that, you know?

(FG14.1)

They [the bosses] called me, they want me to go back. They’re really good to me. Really good people. First, they want me to be well, obviously. But they have to look after their business in there. And they don’t want to replace me. But then they don’t know what’s going to happen. I mean, are they going to get someone short-term? “Are you gonna come back soon? Is it gonna be a really long time?” And I don’t really have answers for them. And I kind of feel bad about that. They’ve been good to me. And I like them. It’s a family type [of business]. They obviously care about my well-being and, it’s just a little family of people working together and so, yeah, it makes me feel bad. They had their vacations scheduled and they were planning on me being there to pick up the slack and maybe work a little more. But this really messed things up. I feel like I let them down.

(FG6.2)

#### Work performance

Those with a job to return to expressed concerns about their ability to perform their work, and were conscious of the judgments that other return-to-work stakeholders (including employer, co-workers and clients) might make, which influenced their RTW expectations.

My biggest concern was how I would perform my job. My concentration is affected. I could probably do [my job] partly and it would be slower. I wouldn’t be that effective, and a lot of what I do is timing; timing’s everything. If I can’t be there at my desk, in my game, playing my best game, then the client’s going to hurt or my performance will be hurt.

(FG13.2)

#### Work characteristics

The duties people were expected to perform and the hours they were required to work also influenced expectations. Generally, these were referenced as a factor complicating the participant’s return to work.

It’s gonna be hard. I don’t know if I’m gonna go back to work because I can’t keep doing the kind of work I was doing [massage therapy], as consistently. And the place I work at, they really don’t give you an option, you don’t get to choose what you do. They just tell you what you’re gonna do. I get no choice.

(FG11.3)

Flexibility in hours and duties was spoken about in terms of facilitating influence.

I have a flex time. […] I have a home office, I can set up in there. I can’t meet clients in my home, but I do have the opportunity to get some stuff done. Um, but with that flex time, that they were hoping for me coming back, like, easing back in. Like, two, three days, you know … it probably would be more receptive than, “You have to be here from 9:00 to 5:00.”

(FG1.2)

#### Physical working conditions

Concerns were expressed about the physical environment and equipment. Generally, these were spoken of as exerting inhibiting forces, extending the estimated time to RTW.

Um, that’s my major fear with [my patient]. […] He should have other care as well, but being so stubborn that he is, you know, it’s just me. And I gotta probably convince him into getting a Hoyer [lift] or something like that, because he doesn’t have [the right equipment].

(FG3.1)

It involves a lot of standing up and like, you know, manual work, and my back just goes crazy when I’m doing that. Before the accident, I was fine, but now, I mean, just standing up now, so it’s just so frustrating. […] It’s hot as hell [in there].

(FG6.2)

#### Occupation and industry

Some occupations, particularly those involving office work, were spoken about in terms of easing the return to work; however, generally, occupation was referenced as a limiting factor in that the participant’s job did not allow for accommodations such as light duties, or was seasonal in nature.

Well, they have, where I work, there’s no light duty, per se. It has to be, you have to be 100%… able to lift 100 pounds. And in my case with [my type of injury], it just takes time to heal.

(FG10.4)

The influence of industry was seen among those who worked in construction. Mainly, this was associated with limitations with the availability of light duties (see above). Industries that were seasonally influenced were also mentioned as delaying return to work.

I’ve been seasonal since I began. [Facilitator: So you won’t be returning until?] Until the start of the season. Because it only goes until about late November, around Thanksgiving. And then you get laid off and start again in April.

(FG8.1)

#### Union job

Participants who were union members appeared less concerned about their return-to-work outcome, with a sentiment along the lines of “When I am ready, they will work it out.” As such, the influence of union representation suggested a reduced urgency for return to work, but that when the participant was ready, union representation would facilitate a positive outcome.

I have a job. We are union. So, I’m all set. So, I don’t question not having a job when I [am ready to] get back. I have the same job as long as I want it.

(FG10.2)

### No job to go back to

For those without a job to return to (n = 7), people referenced their ability to find work. People spoke about how identifying work opportunities and the difficulties they experienced due to losing work because of their work-disability condition influenced their RTW expectations.

#### Work opportunities

People referenced the economy, and the problem of competing with younger workers and workers without a history of time off work.

I’m not foolish. I’m 63 years of age. […] THAT, more than anything else is my biggest question mark because of my age and my injury history. People are gonna be reluctant to hire me.

(FG2.1)

If you’re older and you have stayed out too long and you’re afraid you’re gonna lose your job and you know the market is the size that it is … there’s not supposed to be discrimination but there is.

(FG2.2)

#### Professional networks

Participants without a job to return to also spoke about the time needed to look into what people in their professional networks could do for them.

They told me to go to networking, and I have a really good networking opportunity coming up, so I’m going to be going to that, and I’m going to go to the Women’s Center next week for women that are looking for work.

(FG11.2)

### Considerations for both those with and without a job to return to

#### The “right” job

Finding the “right” job was often discussed, with participants reporting that they wanted to take their time, consider what they could do, and make good decisions (e.g., deciding if they needed to change careers or find a job that would be less likely to aggravate their condition). While this was spoken about by both groups, it was more commonly mentioned by those without a job to return to (71% vs. 30%). Wanting to find the right job was not generally associated with a return to work estimate in the near future, but spoken about in terms of a return-to-work outcome that would be sustainable in the longer term.

What I really want to do, what I’m going to try to do is, and I’ve been applying to the agencies that I mentioned, um, I want to get back into the administrative field—there’s no heavy lifting there.

(FG10.5)

#### Retraining

People spoke about needing retraining, that it had been recommended to them, and that they were considering whether they wanted to undertake retraining or not. Retraining considerations tended to be inhibiting in the short term, and some participants wondered if it would be worth the effort.

I’d really love to go back into a whole different field. But then you’ve gotta take out student loans and all that. And that’s the whole financial thing. It’s like the way the economy is. I know people … you know, went to school and they’re still couldn’t find a job that they went to school for.

(FG11.1)

#### The need for income

Balanced against the individual’s financial resources, was the need for income. This was mentioned as influencing RTW expectations more commonly by persons who were in more tenuous employment with limited access to paid time off and health insurance.

I can’t be out of work. I’m out of vacation time and … I’m out of money, so. That’s where I’m at. If my injury had happened at work, this would be a whole different story. I’d be getting paid to be out of work. I don’t think I’d have a problem, sitting and getting paid and then when I’m better I go back to work. But, I’m not getting paid.

(FG4.4)

The timeframe allowed to find the “right” job and determining if they would pursue retraining tended to be bracketed by income needs.

That’s why I said I gave myself to [later, when benefits run out], because this gave me some time to really look and see what’s out there. I just don’t want to put myself in this position again where I get injured, and it just stopped me from moving forward.

(FG11.1)

## Discussion

Study findings indicate that employees take numerous work-related factors into consideration when estimating their timeframes for returning to work following sickness-related absence. While our earlier study [10] indicted that work-related factors including having a job to go back to, having mixed feelings about returning to work, being concerned about the judgments of return-to-work stakeholders, and feeling needed were influential in the formation of return-to-work expectations, the current, more detailed analyses provide greater insight into the variety of factors considered and how they influenced expected timeframes.

At an overarching level, analyses revealed that the influences mentioned tended to differ depending on whether or not the participant had a job to which they could return. For those who did, the stated influences tended to be more tangible and aligned with findings from the broader return-to-work literature. In particular, participants spoke of the influence of employers / supervisors [12, 15, 16, 19], co-workers [13, 18, 19], union representation [14], work demands and accommodations [11–14]. The finding of limited opportunity for accommodation in the construction industry is also consistent with earlier reports [26]. The impact of the perception of being treated fairly is also consistent with literature describing how perceived injustice might impact disability and rehabilitation outcomes [27, 28]. The impact of worker concerns regarding work performance has received little research attention; however, it has been shown to relate to recovery expectations [3] and longer-term return-to-work success [29]. Results are also in line with previous research findings that show that higher value employees are given more latitude as it relates to time off work and the availability of accommodations [21, 30]. For those without a job to go back to, estimates tended to be less concrete. Inhibiting forces were more typical of those reported in the vocational rehabilitation (VR) literature and included difficulties finding work due to reduced labor market desirability, their history of injury, and advancing age [20]. Such findings suggest that the supports needed by persons without a job to go back to are more akin to those more traditionally provided by those in VR field.

Influences mentioned by both those with a job to go back to and those without focused around finding the right job, retraining and the need for income. Given the potential for longer-term success associated with a good match, it can be argued that supporting seekers in this quest is a worthwhile endeavor. To achieve a good job match, time must be taken to locate the right job, a process that is not all that different from the process for those without musculoskeletal conditions. However, a difference may lie in the fact that for jobseekers with a history of work disability, prospective employers may need to know what supports are needed to enable the work to be done [31]. An additional qualification comes from conventional wisdom indicating that it is easier to get a job when you have a job. As such, those attempting to accomplish an appropriate job match may be advised to support efforts in parallel with a return to a job that is not necessarily the end goal. The need for income adds a further layer of complication to the search for a good job match. The current participants’ desires for “optimal” outcomes were tethered by the practicality of needing income.

In general, more of the influences that people spoke of were referenced as delaying their return to work. While some of these reflected factors inhibiting return to work (e.g. work performance concerns, working conditions, industry and work opportunities), others were delaying return to work in the short term, but not necessarily in the longer term (e.g. union representation, the right job and retraining). In such cases, these influences can be viewed as associated with a return to work that has the potential to be more sustainable in the longer term. There were also some factors that were only spoken of as being catalysts of return to work (e.g. being needed, professional networks and needing income). This finding is consistent with past research indicating that the facilitation of return to work can go beyond the removal of barriers [13]. While, the need for income could be viewed as catalytic of return to work; it can also be argued that returning to work for this reason could be associated with other potentially less favorable outcomes (e.g. returning before physically ready, returning to a job that was likely to result in re-injury / condition exacerbation). As such, it probably should not be viewed as an intervention opportunity. There were also instances where, depending on the participant’s circumstances, the same factor was described as both inhibitory and catalytic of return to work. Examples of this include workplace relationships, work characteristics and occupation. Consistent with current thinking [32–35], such findings stress the importance of understanding the individual’s unique circumstance and tailoring assistance aimed at supporting recovery and return to work to his or her particular needs.

Along with providing an understanding of what work-related factors might be addressed with the aim of improving return-to-work outcomes, study findings have the potential to contribute to the understanding of how to develop work-disability assessment instruments. The “flags system,” which has been used in both practice and research [36], is well developed in terms of physical (red) and psychosocial (yellow) flags; however, workplace perceptions (blue flags) have the potential to benefit from further articulation [37]. The current findings may be helpful in terms of providing a conceptual framework for continuing work involving the identification and measurement of workplace factors impacting work-disability outcomes.

The factors that participants spoke about were consistent with many of those found to relate to other return-to-work outcomes. These similarities suggest that workers use information regarding their work context to construct their estimates. As such, asking a person about their return-to-work expectations and why they have those expectations appears to have the potential to identify opportunities for supporting a safe and timely return to work. In particular, study findings suggest that addressing participant workplace relationships has the potential to improve the injured workers’ outlook, and could be associated with improved outcomes. In addition, helping them to navigate issues of work performance and accommodations appears to have the potential to ease return-to-work concerns. For those without a job to return to, or with apprehensions about returning to their pre-injury workplace, services aimed at helping people make decisions regarding retraining and what job would be suited to their interests and capabilities, within the constraints of financial resources, also appears to have the potential to improve return-to-work expectations.

The current report is an elaboration of our earlier work in which we continued sampling until we felt that we were not accessing new information (reached saturation). While post hoc analysis supported this assessment [10], it may be the case that workers consider additional factors (e.g. attribution of blame). Although it was our assessment that saturation had been achieved, the directionality of influence might need further exploration. For example, in the case of such things as industry, occupation and working conditions, further exploration may reveal that particular circumstances may be associated with the worker feeling more optimistic about their return to work. In order to achieve theoretical saturation, addition work is also needed to determine how influences interact.

A detailed description of study strengths and weakness has been presented previously [10]. To summarize, strengths include a diversity of conditions and comprehensive capture. Limitations include the lack of representation of people who did not expect to return to work, and the fact that all participants were residents of Massachusetts. Additional considerations specific to the current analysis include that the reader interpreting the study findings should be aware of limitations of the study design. Due to the sample size and qualitative nature of the research, we felt it was going beyond the capacity of the method to conduct subgroup analyses. Additional research with larger samples would be needed to address subgroup comparisons and a precise understanding of influence importance. Because participants were not asked specifically about each influence, it may be the case that a factor may have been taken into consideration, but that they just didn’t mention it. As such, counts provide only a limited indication of the importance of influence. We would suggest that future research into this topic be guided by the influences we identified, but employ a quantitative methodology that would allow for the systematic assessment of importance of influences, how influences interact and if this varies based on factors such as participant demographics and condition type.

Study findings indicate a number of opportunities for improving return-to-work expectations through addressing work-related factors. Assessing the relationship between changed return-to-work expectations and actual return to work appears to be a fruitful line of research for both applied and methodological purposes. Another opportunity for further study would be to gain an understanding of 1) the impact of a return-to-work stakeholder inquiring about return-to-work expectations and 2) whether respondents were forthcoming in discussing the factors that influenced their return-to-work expectations when the person they were talking to had a vested interest in the worker’s return to work.

In conclusion, study findings indicate that people reference numerous work-related factors when estimating their timeframes for returning to work, and that many of these have been previously identified as relating to other return-to-work outcomes. The current findings indicate that some work-related factors influence individuals’ expectations such that absent workers consider returning to work *before* being physically and/or mentally ready. Along with needing to return for financial reasons, there appears to be pressure related to fear of job loss and a subsequent inability to find new work. In addition, returning to work has the potential to be delayed due to fears related to job performance and the judgment of employers and co-workers. Findings suggest a number of opportunities to improve return-to-work expectations.
